# Oral Hyaluronan Relieves Wrinkles and Improves Dry Skin: A 12-Week Double-Blinded, Placebo-Controlled Study

**DOI:** 10.3390/nu13072220

**Published:** 2021-06-28

**Authors:** Tzu-Fang Hsu, Zi-Rong Su, Yao-Hao Hsieh, Ming-Fu Wang, Mariko Oe, Ryosuke Matsuoka, Yasunobu Masuda

**Affiliations:** 1 Department of Cosmetic Application, Hung Kuang University, Taichung 433304, Taiwan; tzufang@hk.edu.tw (T.-F.H.); ggg4521tw@gmail.com (Z.-R.S.); dayten0822@gmail.com (Y.-H.H.); 2 Department of Food and Nutrition, Providence University, Taichung 43301, Taiwan; mfwang@pu.edu.tw; 3 R&D Division, Kewpie Corporation, Tokyo 182-0002, Japan; mariko_oe@kewpie.co.jp (M.O.); yasunobu_masuda@kewpie.co.jp (Y.M.)

**Keywords:** hyaluronic acid, dietary supplement, dry skin, wrinkles

## Abstract

Hyaluronan (HA) is present in all connective tissues and organs, including the skin and joint fluid. However, few clinical trials have comprehensively evaluated the impacts of oral HA on skin conditions, including wrinkles and moisturization. In this study, we conducted a placebo-controlled, randomized, double-blind trial of daily HA (120 mg) intake for 12 weeks in 40 healthy Asian men and women (aged 35–64 years). Skin condition was determined by the evaluation of wrinkles, stratum corneum water content, the amount of transepidermal water loss, elasticity, and through image analysis. After 12 weeks, skin condition was significantly improved in terms of wrinkle assessment, stratum corneum water content, transepidermal water loss, and elasticity in the HA group compared to the placebo group. Regarding the percentage change from baseline, wrinkle assessment, stratum corneum water content, and skin elasticity were significantly improved in the HA group versus the placebo group after 8 and 12 weeks of ingestion. The present findings indicate that oral ingestion of HA may suppress wrinkles and improve skin condition.

## 1. Introduction

Hyaluronan (HA) is synthesized in all animals and some microorganisms. In vivo, it is present in all connective tissues and organs, including the skin, joint fluid, blood vessels, serum, the brain, cartilage, heart valves, and the umbilical cord. The skin is the largest organ of the body, accounting for 50% of the total body mass [1].

Under the impact of various factors (e.g., aging, ultraviolet light, and dryness, etc.), the content of HA in the skin becomes low and the water content of the horny layer is decreased, leading to the formation of wrinkles and skin aging [2,3]. A study reported that the amount of HA contained in the skin gradually decreases with aging. More specifically, the content of HA in the skin of individuals aged 75 years is less than a quarter of that measured in persons aged 19 years [4].

Surgical techniques (e.g., injection of HA fillers), topical agents, cosmetics, and health functional foods are currently utilized for the anti-aging treatment of the skin and improvement of dry skin. Surgical techniques offer immediate results; however, they are expensive and associated with a risk of pain and swelling [5]. Various laser and light treatments were also proposed to prevent and treat facial aging and dry skin, with variable results [6,7]. Topical drugs and cosmetic creams are relatively inexpensive treatment options. Nevertheless, they exert limited effects on the site of application and are occasionally linked to the occurrence of skin inflammation due to irritation caused by the preservatives and perfumes contained in the products [8]. Although health functional foods do not have an immediate effect, they are inexpensive; moreover, continuous intake of such foods can offer lasting benefits without unwanted side effects.

It was reported that food components (e.g., collagen peptide, placenta, and HA) function to maintain normal skin conditions, such as stratum corneum water content. However, evidence from randomized, placebo-controlled trials investigating anti-wrinkle therapy did not clearly demonstrate significant improvement in wrinkles by food materials other than HA [9,10,11]. Thus far, multiple skin-moisturizing or anti-wrinkle functions of oral HA ingestion have been reported. Sato et al. investigated 39 Japanese women (aged 37–59 years) with skin dryness, sagging, and wrinkles around the eyes who received HA (120 mg/day) for 6 weeks. After 3 weeks of treatment, the HA group had significantly improved corneal water content compared with the placebo group (*p* < 0.05) [12]. Oe et al. reported that the administration of HA (120 mg/day) for 12 weeks in 50 Japanese men and women (aged 22–59 years) with wrinkles around their eyes resulted in a significant reduction in wrinkle volume ratio after 8 weeks of consumption versus the placebo (*p* < 0.05) [13]. Nevertheless, thus far, no human study has comprehensively evaluated the impacts of HA ingestion on the skin, including skin hydration, skin barrier function, skin elasticity, skin structure, and facial photography. Therefore, we conducted a double-blind, randomized, placebo-controlled, parallel-group trial involving Asian men and women aged 35–64 years to evaluate the impacts of HA consumption on the skin, with a particular emphasis on multifaceted skin assessment.

## 2. Materials and Methods

### 2.1. Study Samples

The subjects received 120 mg/capsule/day of HA (Hyabest^®^(S)LF-P; Kewpie Corporation, Tokyo, Japan) or the placebo (dextrin; Matsutani Chemical Industry Co., Ltd., Hyogo, Japan), taken orally, for 12 consecutive weeks. The HA had 95% purity according to a high-performance liquid chromatography analysis.

### 2.2. Study Design and Ethical Aspects

The study followed a randomized, double-blind, and placebo-controlled design. It was conducted in compliance with the Good Clinical Practice guidelines and the applicable regulatory requirements [14]. Written informed consent was provided by all subjects. The study was registered with the UMIN Clinical Trials Registry (UMIN000043750). In addition, the study was approved by the institutional review board of Antai Medical Care Cooperation Antai Tian-Sheng Memorial Hospital (TSMHIRB 19-021-A) and was conducted in accordance with its rules and regulations. The study protocol conformed to the principles of the Declaration of Helsinki for the use of human subjects in experimental research. The present study was conducted from May to August 2019 in Taichung, Taiwan.

Prior to testing, the volunteers were examined by a cosmetic expert for any serious skin disease or damage, particularly on the cheeks and forearms. Before the study, every volunteer was provided with a volunteer protocol. This protocol, stating the terms and conditions of the testing, was individually signed by each volunteer.

### 2.3. Study Participants

In total, 41 subjects were enrolled into the trial; one subject withdrew from the study for personal reasons. All subjects were aged 35–64 years and met all inclusion criteria. The inclusion criteria were as follows:

(1) Taiwanese male and female subjects aged 30–65 years;
(2) Not currently taking any HA or any other health, nutritional, herbal supplement for skin;
(3) Not currently under-going any medical treatment (including laser treatment, face-lift, or skin-peel);
(4) Not using any topical application cream for skin treatment prescribed by doctors or dermatologists (commonly used facial cleanser, toner, and moisturizer were acceptable).

The subjects were randomly assigned to either the HA or placebo group. They were instructed to maintain their habitual patterns of physical activity throughout the entire study period. All measurements were carried out in a temperature- and relative humidity-controlled room (20 ± 2 °C and 50 ± 5%, respectively).

### 2.4. Study Schedule

The study was conducted over a period of 12 weeks. All parameters were assessed at four observation time points: weeks 0 (baseline), 4, 8, and 12.

### 2.5. Skin Hydration and Barrier Function

Skin hydration was evaluated using a capacitance method (Corneometer^®^ CM 825, Courage+Khazaka Electronic, GmbH, Cologne, Germany). The barrier function was evaluated by measuring transepidermal water loss (TEWL) using the Tewameter^®^ TM300 (Courage+Khazaka Electronic, GmbH). At each time point, measurements were performed at three sites on the face and two sites on the body (i.e., arm and waist). Three recordings were obtained for each of these different areas and the average was calculated.

### 2.6. Skin Elasticity

Skin elasticity was analyzed using the Cutometer^®^ MPA580 (Courage+Khazaka Electronic, GmbH). Skin viscoelastic properties were evaluated by suction and measurements of the consequent skin deformation. This approach measures the elasticity of the upper skin layer using negative pressure, which mechanically deforms the skin. The measurements were performed on the forehead, arm, and waist. For the assessment of body skin elasticity, the Reviscometer^®^ RVM 600 (Courage+Khazaka Electronic, GmbH) was used on the waist. The route resonance of an acoustic wave resonance running time was analyzed, and measurements were conducted at 0°, 90°, 180°, and 360° angles on the arm and waist.

### 2.7. Skin Structure

Ultrasound analysis was used to determine the thickness of the epidermis and dermis, as well as the collagen intensity score. The dermal thickness was measured using a DermaLab^®^ (Cortex Technology, Hadsund, Denmark) ultrasound probe. Measurements were performed at the temple and waist. The skin surface analysis was carried out using the Visioscan VC98 (Courage+Khazaka Electronic, GmbH). A small area of the facial skin (at the crow feet region) was illuminated using non-harmful ultraviolet A light, and a high-resolution image was captured. The image was digitally analyzed using the surface evaluation of living skin method, which provides quantitative measurements of four parameters: roughness (SEr), scaling (SEsc), smoothness (SEsm), and wrinkling (SEw). This system also allows for the evaluation of five additional parameters, namely energy, entropy, homogeneity, variance, and contrast.

### 2.8. Facial Photography

Facial skin was digitally photographed, and the degree of wrinkling on the face was quantitatively analyzed using the VISIA^®^ Evolution ver. 7.0.1 complexion analysis system (Canfield Scientific, Inc., Parsippany, NJ, USA).

### 2.9. Statistical Analysis

It was expected that an evaluable sample of 40 subjects would provide >80% power and an alpha of 0.05 to detect a difference between treatments in the primary outcome variable. The data were expressed as means ± standard deviation. Repeated measures analysis of variance and Dunnett’s test were used to compare the baseline value with that obtained at each measurement time point. After confirming the normal distribution of data using Levene’s test, the independent t-test was used to compare the placebo and HA groups. Statistical tests were performed with a significance level of 5%. SPSS statistics version 25.0 (IBM Corp., Armonk, NY, USA) software was used for the statistical calculations.

## 3. Results

Participants (*n* = 40) were randomized at week 0 and allocated to either the HA (*n* = 20) or placebo (*n* = 20) group. Overall, the baseline demographics and subject characteristics were in line with those of the targeted trial population. All subjects were aged 35–64 years. There was no significant difference between the HA and placebo groups at week 0 (Table 1).

### 3.1. Skin Hydration

Skin moisture over the course of the study is shown in Table 2. The assessment showed that the HA group had significantly higher stratum corneum water content in the facial measurement sites (*p* = 0.02) as compared with the placebo group at 12 weeks after ingestion. The measurements performed on the arm and waist did not show significant differences between the HA and placebo groups. In terms of change from baseline after treatment, the HA group showed a significantly higher percentage change in stratum corneum water content compared with the placebo group at 8 weeks (*p* = 0.01) and 12 weeks (*p* = 0.0003) for the face, 12 weeks (*p* = 0.005) for the arms, and 12 weeks (*p* = 0.005) for the waist. The assessment showed that the HA group had significantly lower transdermal water transpiration in the face versus the placebo group after 12 weeks of treatment (*p* = 0.009). The measurements performed on the arm and waist did not show significant differences between the HA and placebo groups. Higher values of percutaneous water transpiration were associated with rougher and unhealthy skin.

### 3.2. Skin Elasticity

The changes in skin elasticity over time during the study are shown in Table 3 and Table 4. Assessment using Cutometer^®^ MPA580 revealed significantly higher levels of R0, an index of skin acupuncture, in the forehead (*p* = 0.02), arms (*p* = 0.02), and waist (*p* = 0.005) after 12 weeks of ingestion in the HA group compared to the placebo group. The percentage change from the baseline for the waist was significantly higher in the HA group compared with the placebo group for R0 after 8 (*p* = 0.02) and 12 (*p* = 0.000006) weeks of ingestion. In the forehead (*p* = 0.002) and arm (*p* = 0.01), the HA group showed a significantly higher rate of change compared with the placebo group for R0. The HA group did not differ significantly from the placebo group for measurement of skin elasticity as assessed using the Reviscometer^®^ RVM 600.

### 3.3. Skin Structure

The changes in skin architecture over time during the study are shown in Table 5 and Table 6. The HA-treated group did not differ significantly from the placebo-treated group, as assessed using the DermaLab^®^. For thickness at the temple (*p* = 0.02) and waist (*p* = 0.03), the percentage change from baseline after treatment was significantly higher in the HA group than in the placebo group at 12 weeks. The assessment, using Visioscan^®^ VC 98, showed that the HA group had significantly lower levels compared with the placebo group in volume (*p* = 0.048) and variance (*p* = 0.046) after 12 weeks of consumption. After 12 weeks of ingestion, the HA group had significantly higher values for SEsm compared with the placebo group (*p* = 0.02). The percentage change from the baseline was significantly lower in the HA group compared with the placebo group for the volume (*p* = 0.02), SEw (*p* = 0.005), SEr (*p* = 0.04), and variance (*p* = 0.01) after 12 weeks of ingestion. For SEsm, the HA group had a significantly higher rate of change compared with the placebo group after 12 weeks of ingestion (*p* = 0.005).

### 3.4. Facial Photography

**Figure 1:**
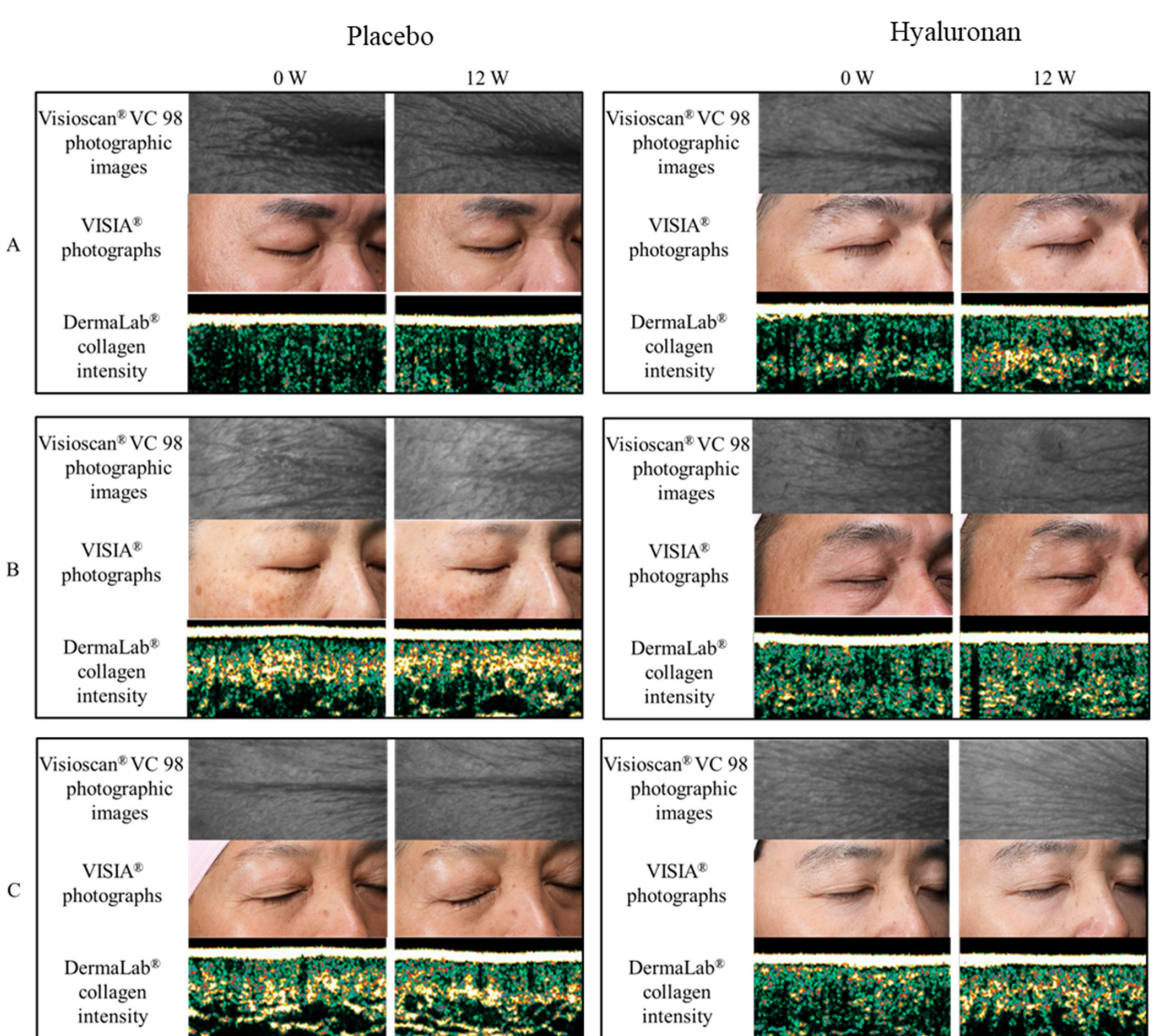
Photographic images of Visioscan^®^ VC 98, VISIA^®^ Photographs, and DermaLab^®^ collagen intensity. Representative images of three subjects from each group are shown (**A**–**C**).

The time course of wrinkles, evaluated using VISIA^®^, during the study is shown in Table 7. There were no significant differences between the HA and placebo groups. Percentage change from baseline after ingestion of HA was significantly improved in the HA group compared with the placebo group at 8 weeks (*p* = 0.02) and 12 weeks (*p* = 0.01). Photographic images are shown in Figure 1.

## 4. Discussion

This study revealed that oral ingestion of HA for 12 weeks significantly improved multiple parameters (e.g., skin moisturization and wrinkles) compared to the placebo. Moreover, there were no adverse events attributable to the consumption of HA. The epidermal turnover was 28 days [15]. Therefore, it was anticipated that the efficacy could be confirmed after 4 weeks of ingestion. However, significant differences were observed between the HA and placebo groups only after 8 weeks of ingestion. In addition to the stratum corneum, dermal fibroblasts also synthesize collagen fibers, elastin fibers, and HA. In vitro, HA stimulates the proliferation of dermal fibroblasts, and HA stimulates in fibroblasts [16,17]. Therefore, it was assumed that 8–12 weeks of ingestion (i.e., a longer period than the epidermal turnover) was required to obtain a statistically significant anti-senescence effect.

This study was conducted on Asian men and women. However, evidence shows that there are no major differences in skin function between sexes or races. Previous studies have reported that there are no significant differences between men and women in stratum corneum water content, TEWL, or skin elasticity, which are key indicators of skin condition [18,19,20,21,22]. Moreover, according to previous studies, there were no significant differences reported in stratum corneum water content between races [23,24,25,26]. Furthermore, investigations did not reveal significant differences in TEWL between black, white, and Hispanic subjects residing in Northern California, USA [27]. Reportedly, there is also no difference in TEWL between Japanese and German individuals [28]. Corneal lipid and ceramide levels were also reported to be similar in Thai and British individuals [29]. In addition, it was shown that the size of corneocytes does not differ greatly between races [30]. In a German study, ingestion of HA significantly improved stratum corneum moisture, elasticity, skin roughness, and wrinkle depth compared with the baseline [31]. Collectively, these studies demonstrate that there are no differences in the effects of HA consumption across races. Therefore, it is inferred that the results of the present study can also be extrapolated in men and women of other races.

Ingested HA is degraded to 4–6 sugars by the gut microbiota and absorbed into the body to reach the skin [32]. Enterobacteriaceae (e.g., Prevotella), which produce hyaluronidases, are widespread in the gut of Asians and Caucasians [33,34,35]. Since there is a constant number of bacteria with HA-degrading capacity in the gut microbiota regardless of race, there is also no major racial difference in the absorption of HA.

The epidermis is composed of the stratum corneum, the granular layer, the stratum spinosum, and the basal layer. HA, which is required in the epidermis, is synthesized in keratinocytes of the basal layer [36,37]. It binds to receptors (CD44) present on the surface of keratinocytes, and normalizes skin function by signaling [38]. It also possesses a high water retention capacity [39]. Furthermore, owing to its skin normalization function and high water retention capacity, it is thought that HA suppresses wrinkle formation in the epidermis. In addition, it is speculated that multiple factors associated with the in vivo functions of HA are involved in the suppression of wrinkle formation. These mechanisms may have led to the improved efficacy and skin condition against wrinkles demonstrated in this study. This study has potential limitations. It was a relatively small sample size study with some indicators, such as SEms, that were not consistent with trends in other indicator results. Further studies, including mechanisms and large-scale clinical studies, are desirable. Skin condition is associated with various components, including diet, sleep, exercise, aging, hormonal balance, ultraviolet radiation, and seasonal variation. It is desirable to review HA intake and lifestyle habits as a whole for maintaining healthy skin. In the future, further investigation on the synergistic anti-aging effect of HA intake and lifestyle habits is warranted.

## 5. Conclusions

This study demonstrated that oral ingestion of HA for 12 weeks may improve wrinkle control and skin condition in healthy Asian men and women aged 35–64 years. Thus, the consumption of HA may be used as a functional food that contributes to the maintenance of skin health.

## Figures and Tables

**Table 1:** Baseline characteristics of subjects.

	Placebo		HA		Total	
	Males	Females	Males	Females	Males	Females
Number	5	15	6	14	11	29
Age (years) mean ± SD	44.2 ± 3.6	44.3 ± 6.5	40.5 ± 4.0	43.5 ± 6.8	43.0 ± 4.1	44.5 ± 6.6
Number	20		20		40	

HA, hyaluronan; SD, standard deviation.

**Table 2:** Measurement of skin hydration using Corneometer^®^ CM825 and transepidermal water loss (TEWL) using Tewameter^®^ TM300.

Evaluation Parameter	Group	Measurement Sites	Week 0	Week 4	Week 8	Week 12
			Mean ± SD	Mean ± SD	Mean ± SD	Mean ± SD
Corneometer	HA	Face	49.5 ± 8.53	51.5 ± 6.55	52.8 ± 7.18 **	54.9 ± 7.06 ***^†^
(AU) ^1^	Placebo	Face	49.1 ± 8.13	49.4 ± 8.79	48.9 ± 9.36	48.8 ± 8.99
	HA	Arm	33.9 ± 8.41	36.8 ± 6.90	36.1 ± 6.38	38.6 ± 7.99 **
	Placebo	Arm	35.4 ± 4.91	36.4 ± 5.78	35.1 ± 4.99	35.6 ± 4.38
	HA	Waist	29.7 ± 7.85	31.9 ± 7.60	30.4 ± 7.20	32.5 ± 7.14 *
	Placebo	Waist	29.7 ± 5.26	31.0 ± 6.57	29.9 ± 6.38	30.2 ± 5.87
Tewameter	HA	Face	12.8 ± 2.39	12.1 ± 1.85	12.0 ± 2.65	10.8 ± 2.49 ***^††^
(g/h/m^2^) ^2^	Placebo	Face	12.6 ± 3.23	12.9 ± 2.67	13.0 ± 3.45	13.3 ± 3.09
	HA	Arm	5.79 ± 1.86	5.45 ± 1.89	5.39 ± 2.10	5.40 ± 2.07
	Placebo	Arm	5.89 ± 2.43	5.94 ±3.06	5.31 ± 2.10	5.57 ± 1.90
	HA	Waist	3.47 ± 2.18	4.27 ± 1.53	3.60 ± 1.88	4.30 ± 1.88
	Placebo	Waist	3.74 ± 2.03	3.41 ± 1.32	3.72 ± 2.68	3.83 ± 1.91

^1^ AU, arbitrary units. ^2^ g/h/m^2^: water lost per hour per square meter of skin. * *p*-values (Dunnett’s test), compared with week 0; * indicates *p* < 0.05, ** indicates *p* < 0.01, and *** indicates *p* < 0.001; ^†^ *p*-values (*t*-test), comparisons between values in the HA and placebo groups; ^†^ indicates *p* < 0.05, and ^††^ indicates *p* < 0.01. HA, hyaluronan; SD, standard deviation.

**Table 3:** Measurement of skin elasticity using Cutometer^®^ MPA580.

Group	Measurement Sites	Evaluation Parameter	Week 0	Week 4	Week 8	Week 12
			Mean ± SD	Mean ± SD	Mean ± SD	Mean ± SD
HA	Forehead	R0	0.09 ± 0.04	0.09 ± 0.04	0.10 ± 0.05	0.11 ± 0.05 **^†^
Placebo			0.08 ± 0.03	0.08 ± 0.03	0.08 ± 0.02	0.08 ± 0.02
HA	Forehead	R1	0.04 ± 0.02	0.04 ± 0.02	0.04 ± 0.01	0.04 ± 0.02
Placebo			0.04 ± 0.02	0.04 ± 0.01	0.04 ± 0.02	0.04 ± 0.01
HA	Forehead	R2	0.62 ± 0.17	0.54 ± 0.14 *	0.52 ± 0.18 **	0.59 ± 0.11
Placebo			0.61 ± 0.14	0.59 ± 0.17	0.55 ± 0.14	0.56 ± 0.15
HA	Forehead	R7	0.44 ± 0.09	0.39 ± 0.09	0.37 ± 0.07 *	0.41 ± 0.12
Placebo			0.43 ± 0.11	0.44 ± 0.16	0.42 ± 0.14	0.42 ± 0.11
HA	Arm	R0	0.24 ± 0.04	0.24 ± 0.04	0.25 ± 0.05	0.26 ± 0.04 *^†^
Placebo			0.23 ± 0.05	0.23 ± 0.06	0.23 ± 0.05	0.22 ± 0.04
HA	Arm	R1	0.03 ± 0.01	0.03 ± 0.01	0.03 ± 0.01	0.03 ± 0.01
Placebo			0.04 ± 0.01	0.03 ± 0.01	0.03 ± 0.01	0.04 ± 0.01
HA	Arm	R2	0.85 ± 0.05	0.86 ± 0.04	0.86 ± 0.04	0.88 ± 0.05
Placebo			0.84 ± 0.05	0.87 ± 0.05	0.86 ± 0.04	0.85 ± 0.05
HA	Arm	R7	0.73±0.09	0.70 ± 0.05	0.72 ± 0.08	0.78 ± 0.06
Placebo			0.74 ± 0.07	0.72 ± 0.08	0.71 ± 0.08	0.75 ± 0.09
HA	Waist	R0	0.32 ± 0.06	0.33 ± 0.07	0.34 ± 0.06	0.38 ± 0.08 ***^††^
Placebo			0.33 ± 0.05	0.31 ± 0.05 **	0.32 ± 0.05	0.32 ± 0.05
HA	Waist	R1	0.04 ± 0.01	0.04 ± 0.01	0.03 ± 0.01 ***	0.04 ± 0.01
Placebo			0.04 ± 0.01	0.04 ± 0.01	0.04 ± 0.01	0.04 ± 0.02
HA	Waist	R2	0.87 ± 0.03	0.88 ± 0.04	0.90 ± 0.04 **	0.89 ± 0.04 *
Placebo			0.87 ± 0.03	0.88 ± 0.03	0.89 ± 0.03	0.88 ± 0.04
HA	Waist	R7	0.77 ± 0.05	0.73 ± 0.07	0.74 ± 0.06	0.78 ± 0.08
Placebo			0.79 ± 0.05	0.75 ± 0.06	0.73 ± 0.07 *	0.77 ± 0.08

R0, skin distension: implication for the firmness of the skin; R1, resilient distension: the ability of the skin to return to its original state; and R2, gross elasticity. * *p*-values (Dunnett’s test), compared with week 0; * indicates *p* < 0.05, ** indicates *p <* 0.01, and *** indicates *p <* 0.001. ^†^ *p*-values (*t*-test), comparisons between values in the HA and placebo groups; ^†^ indicates *p <* 0.05, and ^††^ indicates *p <* 0.01. HA, hyaluronan; SD, standard deviation.

**Table 4:** Measurement of skin elasticity using Reviscometer^®^ RVM 600.

Group	Week 0	Week 4	Week 8	Week 12
	Mean ± SD	Mean ± SD	Mean ± SD	Mean ± SD
HA	269 ± 9.64	267 ± 9.85	269 ± 9.33	269 ± 11.6
Placebo	269 ± 12.19	269 ± 12.5	270 ± 11.7	269 ± 11.1

Resonance running times for angles 0°, 45°, 90°, and 135°. HA, hyaluronan; and SD, standard deviation.

**Table 5:** Measurement of skin structure using DermaLab^®^.

Group	Measurement Sites	Evaluation Parameter	Week 0	Week 4	Week 8	Week 12
			Mean ± SD	Mean ± SD	Mean ± SD	Mean ± SD
HA	Temple	Thickness	1152 ± 195	1151 ± 205	1177 ± 204	1207 ± 217 *
Placebo	Temple	Thickness	1152 ± 229	1152 ± 225	1151 ± 210	1150 ± 212
HA	Waist	Thickness	1537 ± 302	1544 ± 280	1566 ± 313	1614 ± 303 **
Placebo	Waist	Thickness	1513 ± 259	1501 ± 254	1509 ± 275	1517 ± 257
HA	Temple	Intensity	46.6 ± 9.96	47.9 ± 11.9	49.6 ± 13.6	52.0 ± 12.3 **
Placebo	Temple	Intensity	47.8 ± 11.9	47.0 ± 10.8	47.3 ± 11.4	48.7 ± 11.4
HA	Waist	Intensity	38.4 ± 13.4	37.6 ± 13.0	38.8 ± 13.8	41.8 ± 13.5 **
Placebo	Waist	Intensity	37.8 ± 12.8	39.8 ± 12.8	38.1 ± 12.6	39.3 ± 12.7

* *p*-values (Dunnett’s test) compared with week 0; * indicates *p* < 0.05, and ** indicates *p* < 0.01. HA, hyaluronan; and SD, standard deviation.

**Table 6:** Measurement of skin micro-relief parameters using Primo and Visioscan^®^ VC 98.

Group	Evaluation Parameter	Week 0	Week 4	Week 8	Week 12
		Mean ± SD	Mean ± SD	Mean ± SD	Mean ± SD
HA	Surface	408 ± 54.0	429 ± 54.0	423 ± 58.5	432 ± 47.8
Placebo		422 ± 38.0	430 ± 43.4	421 ± 44.2	427 ± 45.2
HA	Volume	69.2 ± 22.7	64.7 ± 26.5	59.6 ± 20.2	52.1 ± 9.29 **^†^
Placebo		60.5 ± 19.0	61.1 ± 15.7	58.7 ± 13.2	59.8 ± 14.1
HA	SEw	115 ± 23.1	110 ± 26.6	103 ± 33.9	97.8 ± 31.0 **
Placebo		115 ± 36.9	116 ± 34.7	113 ± 30.8	116 ± 28.7
HA	SEsc	0.52 ± 0.13	0.49 ± 0.12	0.45 ± 0.09	0.43 ± 0.11 **
Placebo		0.47 ± 0.13	0.45 ± 0.13	0.44 ± 0.07	0.42 ± 0.11
HA	SEsm	255 ± 48.9	254 ± 45.5	266 ± 48.2	287 ± 43.4 *^†^
Placebo		252 ± 62.4	241 ± 64.5	252 ± 59.6	245 ± 60.7
HA	SEr	3.63 ± 1.42	3.59 ± 1.43	3.51 ± 1.56	3.25 ± 1.39
Placebo		3.65 ± 1.44	3.62 ± 1.43	3.56 ± 1.40	3.66 ± 1.29
HA	NRJ	0.03 ± 0.007	0.03 ± 0.008	0.03 ± 0.008	0.03 ± 0.007
Placebo		0.02 ± 0.005	0.02 ± 0.004	0.03 ± 0.005	0.03 ± 0.003
HA	ENT	1.52 ± 0.04	1.52 ± 0.04	1.52 ± 0.04	1.52 ± 0.04
Placebo		1.51 ± 0.03	1.51 ± 0.02	1.51 ± 0.03	1.51 ± 0.02
HA	HOM	1.40 ± 0.06	1.40 ± 0.08	1.42 ± 0.07	1.42 ± 0.08
Placebo		1.39 ± 0.05	1.40 ± 0.05	1.40 ± 0.05	1.40 ± 0.05
HA	CONT	1.03 ± 0.28	1.00 ± 0.32	0.99 ± 0.30	0.95 ± 0.32 *
Placebo		0.99 ± 0.24	0.90 ± 0.13	0.90 ± 0.20 *	0.95 ± 0.17
HA	VAR	3.83 ± 0.79	3.75 ± 0.80	3.59 ± 0.73	3.34 ± 0.63 **^†^
Placebo		3.74 ± 0.47	3.67 ± 0.49	3.48 ± 0.51 *	3.70 ± 0.46

SD, standard deviation; HA, hyaluronan; SEw, wrinkles: higher values indicated more wrinkles; SEsc, scaliness: lower values indicated less desquamation on the stratum corneum and less scaliness of the skin; SEsm, skin smoothness: lower values indicated smoother skin; SEr, skin roughness: lower values indicated rougher skin; NRJ, energy; ENT, entropy; HOM, homogeneity: higher in young, smooth skin; CONT, contrast; and VAR, variance: lower in young, smooth skin. * *p*-values (Dunnett’s test), compared with week 0; * indicates *p* < 0.05, and ** indicates *p* < 0.01. ^†^ *p*-values (*t*-test), comparisons between values in the HA and placebo groups; ^†^ indicates *p* < 0.05.

**Table 7:** Skin image by VISIA^®^.

Group	Evaluation Parameter	Week 0	Week 4	Week 8	Week 12
		Mean ± SD	Mean ± SD	Mean ± SD	Mean ± SD
HA	Wrinkle	192 ± 38.0	187 ± 38.8	188 ± 38.7	184 ± 39.2 **
Placebo	Wrinkle	197 ± 42.6	202 ± 41.2	206 ± 37.4	204 ± 41.9

** indicates *p* < 0.01 (Dunnett’s test), compared with week 0. SD, standard deviation; and HA, hyaluronan.

## Data Availability

The data presented in this study are available on request from the corresponding author. The data are not publicly available due to ethical restrictions.
